# A Two-Stage PPO–RLMPA Framework for Dynamic Economic Dispatch with Renewable Energy and Storage Integration

**DOI:** 10.3390/biomimetics11060400

**Published:** 2026-06-06

**Authors:** Kemal Keskin

**Affiliations:** 1 Department of Transport and Planning, Delft University of Technology, 2628 CN Delft, The Netherlands; k.keskin@tudelft.nl; 2 Department of Electrical and Electronics Engineering, Eskisehir Osmangazi University, Eskisehir 26040, Turkey

**Keywords:** dynamic economic dispatch, RLMPA, deep reinforcement learning, Proximal Policy Optimization, Marine Predators Algorithm, Deep Q-Network, Hybrid Optimization, Renewable Energy Integration, pumped-storage hydro

## Abstract

The Dynamic Economic Dispatch (DED) problem underpins the cost-efficient and reliable operation of modern power systems, yet valve-point loading, ramp-rate coupling, and the growing share of intermittent wind, photovoltaic, and pumped-storage hydro (PSH) resources render it highly non-convex. Metaheuristic methods typically require large computational budgets and hand-crafted constraint-handling rules, whereas deep reinforcement learning agents rarely guarantee the feasibility of the schedules they produce. To address both limitations, this paper proposes a Two-Stage PPO–RLMPA framework that couples data-driven policy learning with a biomimetic metaheuristic search inspired by marine predator–prey dynamics. In the first stage, a Proximal Policy Optimization (PPO) agent is trained on a Markov Decision Process reformulation of DED in which a deterministic Safety Layer projects every raw action onto the feasible set defined by capacity, ramp-rate, and power-balance constraints, so the policy only observes physically viable transitions. In the second stage, the PPO dispatch is refined by the RLMPA module, a Marine Predators Algorithm (MPA) whose exploration–exploitation balance, Lévy-flight foraging, and Fish Aggregating Devices (FADs) attraction mechanisms emulate strategies documented in marine ecosystems; its step-size factor and FADs probability are further adapted online by a Deep Q-Network. This biomimetics-informed refinement translates predator–prey foraging intelligence into economically efficient thermal dispatch under valve-point non-convexity. Across 30 independent runs on ten- and twenty-unit benchmark systems with wind, PV, and PSH integration, the framework attains best costs of USD 368,763 and USD 737,348 on Test Systems 1 and 2, corresponding to reductions of approximately 1.1% and 4.4% over the CFCEP baseline, with zero post-repair constraint violations in every run.

## 1. Introduction

The Economic Dispatch (ED) problem is a fundamental operational task in modern power systems, aiming to allocate the total electricity demand among the available generating units in a way that minimizes the total operating cost while respecting physical and network constraints [1]. As networks evolve toward higher renewable penetration and more flexible demand patterns, the static formulation of ED has been extended to the Dynamic Economic Dispatch (DED) problem, which determines an optimal generation schedule over a scheduling horizon subject to ramp-rate limits and time-coupled operational constraints.

Various metaheuristic methods has been proposed to address DED. Particle swarm optimization and its variants have been applied to single- and multi-objective dispatch formulations [2,3], evolutionary programming has demonstrated strong performance in non-convex settings with valve-point loading [4,5,6], and nondominated-sorting genetic algorithms have been used for emission-aware multi-objective dispatch [7,8]. Differential evolution variants with adaptive mutation strategies [9,10], bacterial foraging [11], harmony search [12], and more recent nature-inspired algorithms such as improved grey wolf [13], advanced kernel search [14], and bottlenose dolphin optimizers [15] have further broadened the metaheuristic toolbox. Although these techniques improve global exploration and convergence robustness, their reliance on population-based search typically leads to high computational cost and requires carefully engineered constraint-handling routines to ensure feasibility.

Among biomimetic optimizers, the Marine Predators Algorithm (MPA) is explicitly grounded in the foraging ecology of apex marine predators [16]. MPA models three hunting phases that transition with a velocity ratio: a Brownian-motion regime for wide exploration, a mixed Brownian–Lévy regime for adaptive search, and a Lévy-flight exploitation regime for fine local polishing. An additional Fish Aggregating Devices (FADs) operator simulates attraction to resource hotspots, helping the population escape deceptive local minima. Such mechanisms are particularly relevant to Dynamic Economic Dispatch with valve-point loading, where the cost surface exhibits prohibited operating zones and numerous local optima. The present work adopts this biomimetic core in Stage 2 of a hybrid framework, while Stage 1 supplies a fast, constraint-safe policy through deep reinforcement learning; together, they form a biomimetics–learning co-design for renewable-integrated dispatch.

The integration of intermittent renewable sources and energy-storage technologies has added new layers of complexity to DED. Wind and solar generation introduce stochasticity that cannot be fully captured by purely deterministic formulations, while pumped-storage hydro (PSH) units introduce inter-temporal couplings through reservoir dynamics [17]. Several studies have accordingly proposed DED formulations that explicitly couple thermal, wind, PV, and PSH resources [17,18,19,20], confirming that hybrid energy systems substantially increase the dimensionality of the underlying optimization problem.

To address forecast uncertainty, a number of works have proposed scenario-based and fuzzy-adaptive formulations, often embedded within hybrid metaheuristic frameworks [21,22,23,24]. While these approaches improve robustness against renewable variability, they tend to further increase the computational burden, which in turn motivates the development of faster and more adaptive solution schemes.

Deep reinforcement learning (DRL) has recently emerged as a complementary paradigm, reformulating DED as a sequential decision-making problem. In this view, dispatch decisions are modeled as a Markov Decision Process in which actions at each time step influence the future evolution of the system. Policy-gradient methods such as Proximal Policy Optimization (PPO) have been successfully applied to economic dispatch and to closely related operational-scheduling tasks. Rizki et al. [25] train a PPO agent on a 15-generator dispatch problem with ramp-rate and reserve constraints, while Liu et al. [26] employ PPO with a clipped surrogate objective for automatic-generation-control dynamic optimization under renewable variability. Deep-deterministic-policy-gradient variants have also been proposed for renewable-rich dispatch: Zhu et al. [27] adopt a DDPG agent for the scheduling of a wind-dominated distribution network, reducing power-fluctuation metrics on IEEE benchmarks. Despite these advances, a pure DRL policy rarely guarantees strict feasibility of the schedules it produces, and its global optimality on highly multimodal landscapes such as those induced by valve-point loading is not ensured. These limitations have motivated two complementary research directions. First, safe or constraint-aware reinforcement learning enforces operational limits by projecting the action onto the feasible set or by augmenting the policy with a differentiable safety layer [28]. Second, hybrid learning–metaheuristic approaches combine the fast inference of a trained policy with the global search capability of a population-based optimizer, as recently demonstrated by Gurumoorthi et al. [29], who couple a policy network with a quantum-inspired genetic algorithm for optimal power flow. Despite their promise, these two directions have largely been pursued independently, and their benefits for renewable- and storage-integrated DED remain unexplored.

Building on both directions simultaneously, this paper proposes the Two-Stage PPO-RLMPA framework, which integrates constraint-aware policy learning with an adaptively tuned metaheuristic refinement. In the first stage, a Proximal Policy Optimization agent interacts with a Markov Decision Process reformulation of DED through an environment that embeds a deterministic Safety Layer, enforcing capacity, ramp-rate, and power-balance constraints at every time step. In the second stage, a Marine Predators Algorithm whose step-size factor and Fish Aggregating Devices probability are tuned online by a Deep Q-Network (hereafter referred to as the RLMPA) module performs a localized, constraint-aware refinement of the PPO dispatch [30,31]. This combination exploits the sub-second inference of a trained policy and the global exploration strength of a metaheuristic while guaranteeing the feasibility of every generated schedule. The main contributions of this study are as follows:

1. The DED problem is reformulated as a Markov Decision Process with a state vector of dimension $7+N_{G}$, yielding 17- and 27-dimensional observations for the 10- and 20-unit benchmark systems, respectively. The MDP is solved by a PPO agent that produces a full 24 h feasible schedule in well under one second at inference once the policy is trained offline.
2. A deterministic Safety Layer is embedded inside the reinforcement learning environment to project every raw action onto the feasible set defined by capacity, ramp-rate, and power-balance constraints. The layer guarantees fully feasible transitions at every time step and removes the need for arbitrary penalty terms in the reward signal.
3. The Two-Stage PPO-RLMPA framework is introduced. Stage 1 provides a feasible near-optimal baseline through PPO with the Safety Layer; Stage 2 employs the RLMPA module to perform a localized refinement around the PPO baseline. Using the PPO schedule as the elite initialization avoids the curse of dimensionality observed when classical metaheuristics are started from random points in the 240- and 480-dimensional search spaces of the two benchmarks.
4. The framework is evaluated on two DED benchmark systems against standalone PPO, classical MPA, DQN-MPA, and PSO over 30 independent runs. It attains the lowest mean and best operating costs reported on both systems, with cost reductions of approximately 1.1% and 4.4% relative to the CFCEP baseline reported by Basu [17], and reports zero average post-repair constraint violation in every run. Stage 1 inference is sub-second; the full two-stage pipeline including optional RLMPA refinement requires roughly 20–22 s per horizon and targets offline day-ahead dispatch.

## 2. Problem Formulation

**Figure 1:**
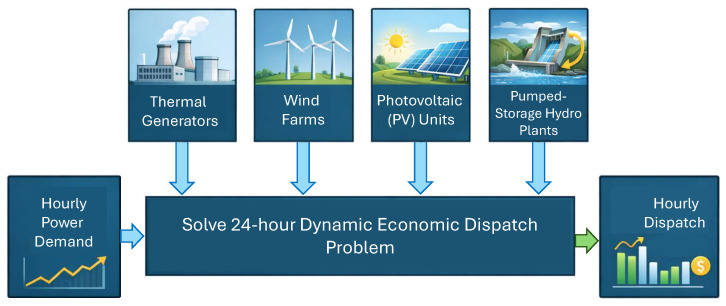
Block diagram of Dynamic Economic Dispatch Problem.

This study considers the dynamic economic dispatch (DED) problem of a hybrid power generation system composed of thermal generators, wind farms, photovoltaic (PV) units, and pumped-storage hydro (PSH) plants (See Figure 1).

The objective is to determine the optimal generation schedule of all participating units over a predefined scheduling horizon while satisfying system demand and operational constraints. Due to the intermittent nature of renewable energy sources and the storage capability of PSH units, the dispatch problem becomes strongly time-coupled and nonlinear. Therefore, an accurate mathematical formulation is essential for representing both system flexibility and operational limitations.

### 2.1. Objective Function

The main objective of the proposed optimization problem is to minimize the total operating cost of the hybrid generation system over the entire scheduling period. The total cost consists of fuel costs associated with thermal units and operational costs of renewable generators.

The overall objective function is defined as

(1) $$J=\sum_{t=1}^{T}\left[\sum_{i=1}^{N_{G}}C_{i}\left(P_{i}\left(t\right)\right)+\sum_{w=1}^{N_{W}}c_{w}^{W}\;W_{w}\left(t\right)+\sum_{m=1}^{N_{S}}c_{m}^{S}\;S_{m}\left(t\right)\right]$$

where *T* denotes the number of dispatch intervals. The variables $P_{i}\left(t\right)$, $W_{k}\left(t\right)$, and $S_{m}\left(t\right)$ represent the power outputs of thermal generators, wind units, and PV plants at time interval *t*, respectively. Parameters $c_{w}^{W}$ and $c_{m}^{S}$ denote the cost coefficients of wind and PV generation, respectively.

The fuel cost function of each thermal unit is modeled using a quadratic function augmented with a sinusoidal term to capture the valve-point loading effect, which introduces nonconvexity into the optimization problem. The cost function of generator *i* is therefore expressed as

(2) $$C_{i}\left(P_{i}\left(t\right)\right)=\alpha_{i}+\beta_{i}P_{i}\left(t\right)+\chi_{i}P_{i}^{2}\left(t\right)+\delta_{i}\left|\sin\left(\zeta_{i}\left(P_{i}^{\min}-P_{i}\left(t\right)\right)\right)\right|$$

This formulation improves modeling accuracy compared with conventional quadratic cost representations by reflecting the rippling behavior of the steam admission valves.

### 2.2. Power Balance Constraint

For secure system operation, the total generated power must satisfy the load demand at each dispatch interval while accounting for transmission losses. Since the PSH unit can operate in generating mode, pumping mode, or idle mode, three different power balance relationships are considered.

When the PSH unit operates in generating mode, it contributes power to the grid, and the balance equation becomes

(3) $$\sum_{i=1}^{N_{G}}P_{i}\left(t\right)+\sum_{w=1}^{N_{W}}W_{w}\left(t\right)+\sum_{m=1}^{N_{S}}S_{m}\left(t\right)+\sum_{j=1}^{N_{H}}H_{j}\left(t\right)=D\left(t\right)+L\left(t\right)$$

When the PSH unit operates in pumping mode, it absorbs electrical energy from the system and stores it as potential energy in the upper reservoir. In this case, the pumping power must be included on the demand side of the balance equation:

(4) $$\sum_{i=1}^{N_{G}}P_{i}\left(t\right)+\sum_{w=1}^{N_{W}}W_{w}\left(t\right)+\sum_{m=1}^{N_{S}}S_{m}\left(t\right)=D\left(t\right)+L\left(t\right)+\sum_{j=1}^{N_{H}}U_{j}\left(t\right)$$

When the PSH unit remains idle, it neither generates nor consumes power, and the conventional power balance equation applies:

(5) $$\sum_{i=1}^{N_{G}}P_{i}\left(t\right)+\sum_{w=1}^{N_{W}}W_{w}\left(t\right)+\sum_{m=1}^{N_{S}}S_{m}\left(t\right)=D\left(t\right)+L\left(t\right)$$

These three operating modes enable the PSH system to provide flexibility for balancing renewable intermittency.

### 2.3. Transmission Loss Model

Transmission losses are represented using the classical *B*-coefficient loss formula, which expresses network losses as a quadratic function of generator outputs. This formulation is widely used in economic dispatch studies due to its simplicity and acceptable accuracy.

The transmission loss at time interval *t* is calculated as

(6) $$L\left(t\right)=\sum_{r=1}^{N_{T}}\sum_{s=1}^{N_{T}}P_{r}\left(t\right)B_{rs}P_{s}\left(t\right)+\sum_{r=1}^{N_{T}}B_{r0}P_{r}\left(t\right)+B_{00}$$

where $N_{T}$ denotes the total number of generating units participating in the dispatch process.

### 2.4. Wind Power Generation Model

Wind generation output depends strongly on wind speed variations and turbine operating characteristics. Therefore, a piecewise nonlinear model is adopted to represent the relationship between wind speed and generated power.

The output power of wind generator *w* is defined as

(7) $$W_{w}\left(t\right)=\left\{\begin{array}{cc} 0\; & v_{w}\left(t\right)<v_{w}^{\mathrm{in}}\\ \left(a_{w}+b_{w}v_{w}\left(t\right)+c_{w}v_{w}^{2}\left(t\right)\right)W_{w}^{\mathrm{rated}}\; & v_{w}^{\mathrm{in}}\leq v_{w}\left(t\right)<v_{w}^{\mathrm{rated}}\\ W_{w}^{\mathrm{rated}}\; & v_{w}^{\mathrm{rated}}\leq v_{w}\left(t\right)\leq v_{w}^{\mathrm{out}}\\ 0\; & v_{w}\left(t\right)>v_{w}^{\mathrm{out}} \end{array}\right.$$

The coefficients $a_{w}$, $b_{w}$, and $c_{w}$ appearing in the intermediate wind-speed region are obtained by enforcing continuity conditions on the wind turbine power curve between the cut-in and rated wind speeds. Specifically, the generated power is assumed to be zero at the cut-in speed and equal to the rated output at the rated wind speed.

Accordingly, the coefficients are calculated as

(8) $$a_{w}=\frac{W_{w}^{\mathrm{rated}}v_{w}^{\mathrm{in}}v_{w}^{\mathrm{rated}}}{{\left(v_{w}^{\mathrm{rated}}-v_{w}^{\mathrm{in}}\right)}^{2}}$$

(9) $$b_{w}=\frac{-W_{w}^{\mathrm{rated}}\left(v_{w}^{\mathrm{rated}}+v_{w}^{\mathrm{in}}\right)}{{\left(v_{w}^{\mathrm{rated}}-v_{w}^{\mathrm{in}}\right)}^{2}}$$

(10) $$c_{w}=\frac{W_{w}^{\mathrm{rated}}}{{\left(v_{w}^{\mathrm{rated}}-v_{w}^{\mathrm{in}}\right)}^{2}}$$

This formulation captures the turbine cut-in, rated-output, and cut-out operating regions, providing a realistic approximation of wind generation behavior.

### 2.5. Solar PV Generation Model

The output power of PV units depends on both solar irradiance and ambient temperature conditions. To capture these environmental effects, the following linearized PV generation model is adopted:

(11) $$S_{m}\left(t\right)=S_{m}^{\mathrm{rated}}\left[1-\kappa \left(T_{\mathrm{amb}}\left(t\right)-T_{\mathrm{ref}}\right)\right]\frac{G(t)}{G_{\mathrm{std}}}$$

Here, κ>0 is the PV temperature derating coefficient and $G_{\mathrm{std}}=1000$ W/m^2^ is the standard test-condition irradiance. This expression reflects the sensitivity of PV efficiency to temperature variations while maintaining computational simplicity.

### 2.6. Reservoir Dynamics of the PSH System

The PSH unit plays an essential role in compensating renewable variability by storing surplus energy and supplying power during peak demand periods. Therefore, reservoir volume dynamics must be explicitly modeled.

During pumping operation:

(12) $$V_{j}\left(t+1\right)=V_{j}\left(t\right)+\phi_{j}U_{j}\left(t\right)$$

During generating operation:

(13) $$V_{j}\left(t+1\right)=V_{j}\left(t\right)-\psi_{j}H_{j}\left(t\right)$$

During idle operation:

(14) $$V_{j}\left(t+1\right)=V_{j}\left(t\right)$$

Reservoir storage limits are enforced as

(15) $$V_{j}^{\min}\leq V_{j}\left(t\right)\leq V_{j}^{\max}$$

Additionally, the initial and final reservoir levels are assumed identical to ensure cyclic operation across the scheduling horizon:

(16) $$V_{j}\left(0\right)=V_{j}\left(T\right)$$

### 2.7. Thermal Generator Operating Constraints

Thermal generators are subject to physical operating limits that must be satisfied throughout the scheduling horizon.

First, generation capacity limits are imposed as

(17) $$P_{i}^{\min}\leq P_{i}\left(t\right)\leq P_{i}^{\max}$$

Furthermore, ramp-rate limits restrict rapid variations in generator output between consecutive time intervals:

(18) $$P_{i}\left(t\right)-P_{i}\left(t-1\right)\leq R_{i}^{↑}$$

(19) $$P_{i}\left(t-1\right)-P_{i}\left(t\right)\leq R_{i}^{↓}$$

These constraints ensure secure and feasible operation of thermal generation units under practical operating conditions.

## 3. Proposed Methodology

To overcome the computational burden and constraint-handling limitations of purely metaheuristic approaches, this paper introduces a two-stage hybrid framework that couples a deep reinforcement learning (DRL) policy with a reinforcement-learning-tuned metaheuristic refinement. The first stage employs a Proximal Policy Optimization (PPO) agent operating on a Markov Decision Process reformulation of the Dynamic Economic Dispatch (DED) problem, augmented by a deterministic Safety Layer that guarantees constraint satisfaction at every timestep. The second stage performs a localized refinement of the PPO baseline through a Marine Predators Algorithm (MPA) whose search parameters are adaptively tuned by a Deep Q-Network (DQN) controller.

### 3.1. MDP Formulation for DED

The DED problem is reformulated as a Markov Decision Process (MDP) defined by the tuple M=(S,A,P,R,γ), where S is the state space, A is the action space, $P(s_{t+1}|s_{t},a_{t})$ is the transition kernel induced by the grid dynamics and the embedded Safety Layer, R is the scalar reward, and γ∈(0,1) is the discount factor.

State space: The state vector at hour *t* captures both the exogenous grid conditions and the previous operating point of the thermal fleet:

(20) $$s_{t}={\left[\;\frac{t}{T},\;\tilde{D}\left(t\right),\;\tilde{W}\left(t\right),\;\tilde{S}\left(t\right),\;{\tilde{P}}_{\mathrm{psh}}\left(t\right),\;{\tilde{T}}_{\mathrm{amb}}\left(t\right),\;\rho \left(t\right),\;{\hat{P}}_{1}\left(t-1\right),\ldots ,{\hat{P}}_{N_{G}}\left(t-1\right)\;\right]}^{\;⊤}$$

where the tilde denotes a normalized quantity scaled by the nominal capacity of the associated resource, ρ(t) is the required thermal share which is defined as follows:

(21) $$\rho \left(t\right)=\left(D\left(t\right)-\;\sum_{w=1}^{N_{W}}\;W_{w}\left(t\right)-\;\sum_{m=1}^{N_{S}}\;S_{m}\left(t\right)-\;\sum_{j=1}^{N_{H}}\;H_{j}\left(t\right)\right)/\;\sum_{i=1}^{N_{G}}\;P_{i}^{\max}$$

and ${\hat{P}}_{i}\left(t-1\right)$ is the normalized output of thermal unit *i* at the previous hour and calculated as follows:

(22) $${\hat{P}}_{i}\left(t-1\right)=\left(P_{i}\left(t-1\right)-P_{i}^{\min}\right)/\left(P_{i}^{\max}-P_{i}^{\min}\right)$$

here, ${\tilde{P}}_{\mathrm{psh}}\left(t\right)$ denotes the net PSH contribution at hour *t*, obtained by normalizing $P_{\mathrm{psh}}\left(t\right)=\sum_{j}H_{j}\left(t\right)$ during generation and $P_{\mathrm{psh}}\left(t\right)=-\sum_{j}U_{j}\left(t\right)$ during pumping by the aggregated PSH capacity. The resulting dimensionality is $7+N_{G}$, which yields 17 for Test System 1 and 27 for Test System 2.

Action space: The policy outputs a continuous vector $a_{t}\in {\left[-1,1\right]}^{N_{G}}$ whose components are affinely mapped to the dynamically feasible interval of each unit:

(23) $${\underline{P}}_{i}\left(t\right)=\max\;\left(P_{i}^{\min},\;P_{i}\left(t-1\right)-R_{i}^{↓}\right),\;{\bar{P}}_{i}\left(t\right)=\min\;\left(P_{i}^{\max},\;P_{i}\left(t-1\right)+R_{i}^{↑}\right).$$

A raw action $a_{t,i}$ is mapped to a provisional dispatch

(24) $${\tilde{P}}_{i}\left(t\right)={\underline{P}}_{i}\left(t\right)+\frac{a_{t,i}+1}{2}\left({\bar{P}}_{i}\left(t\right)-{\underline{P}}_{i}\left(t\right)\right),$$

which is subsequently corrected by the Safety Layer of Section 3.3 to guarantee the power-balance equality.

Reward: The reward promotes cost minimization and is defined as a normalized negative instantaneous cost:

(25) $$r_{t}=-\frac{1}{C_{\mathrm{norm}}}\;\left[\sum_{i=1}^{N_{G}}C_{i}\;\left(P_{i}\left(t\right)\right)+\sum_{w=1}^{N_{W}}c_{w}^{W}\;W_{w}\left(t\right)+\sum_{m=1}^{N_{S}}c_{m}^{S}\;S_{m}\left(t\right)\right]$$

with $C_{\mathrm{norm}}=10^{4}$ to keep the return within a numerically stable range during gradient updates. Since the Safety Layer strictly enforces both ramp-rate and power-balance constraints, no penalty term is injected into (25); the agent only experiences costs of physically viable transitions.

### 3.2. Proximal Policy Optimization (PPO) Agent

PPO is an on-policy actor–critic algorithm that maximizes a clipped surrogate objective, combining the sample efficiency of trust-region methods with the simplicity of first-order gradient updates. Let $\pi_{\theta }\left(a|s\right)$ denote the stochastic policy parameterized by a neural network with parameters θ, and $V_{\phi }\left(s\right)$ its value function. Given a batch of trajectories collected under the previous policy $\pi_{\theta_{\mathrm{old}}}$, PPO updates θ by maximizing

(26) $$L^{\mathrm{CLIP}}\left(\theta \right)=E_{t}\;\left[\min\;\left(\rho_{t}\left(\theta \right)\;{\hat{A}}_{t},\;\mathrm{clip}\left(\rho_{t}\left(\theta \right),\;1-\epsilon ,\;1+\epsilon \right){\hat{A}}_{t}\right)\right].$$

where $\rho_{t}\left(\theta \right)$ is the probability ratio:

(27) $$\rho_{t}\left(\theta \right)=\pi_{\theta }\left(a_{t}|s_{t}\right)/\pi_{\theta_{\mathrm{old}}}\left(a_{t}|s_{t}\right).$$

and ${\hat{A}}_{t}$ denotes the Generalized Advantage Estimation (GAE):

(28) $${\hat{A}}_{t}=\sum_{l=0}^{T-t-1}{\left(\gamma \lambda \right)}^{l}\;\delta_{t+l},\;\delta_{t}=r_{t}+\gamma V_{\phi }\left(s_{t+1}\right)-V_{\phi }\left(s_{t}\right).$$

The total loss augments $L^{\mathrm{CLIP}}$ with a value-function loss and an entropy bonus that encourages exploration:

(29) $$L\left(\theta ,\phi \right)=-L^{\mathrm{CLIP}}\left(\theta \right)+c_{1}\;E_{t}\;\left[{\left(V_{\phi }\left(s_{t}\right)-R_{t}\right)}^{2}\right]-c_{2}\;E_{t}\;\left[H\left(\pi_{\theta }\left(\cdot |s_{t}\right)\right)\right].$$

Both actor and critic are implemented as multilayer perceptrons with 2 hidden layers of 256 units and tanh activations. In total, 4 parallel environments collect rollouts of $n_{\mathrm{steps}}=192$ transitions each, which are split into mini-batches of size 48 and used for 10 epochs of stochastic gradient ascent with the Adam optimizer. The hyper-parameters are γ=0.99, λ=0.95, ϵ=0.2, learning rate $3\times 10^{-4}$, value-loss coefficient $c_{1}=0.5$, and entropy coefficient $c_{2}=0.01$. Convergence is monitored every 5000 timesteps through a callback that re-evaluates the policy on the full 24 h horizon and checkpoints the model with the lowest average cost.

### 3.3. Safety Layer

Raw actor outputs may violate the physical constraints of the grid whenever the policy is only partially trained or the load signal changes abruptly. To eliminate such infeasible transitions, the proposed environment integrates a deterministic Safety Layer between the action output and the reward computation. The layer operates on three principles: (i) action-to-feasible interval mapping, (ii) ramp-aware clipping, and (iii) proportional redistribution that enforces the hourly power balance.

Given the provisional dispatch of Equation (24), the residual power mismatch at time *t* is

(30) $$\Delta \left(t\right)=D\left(t\right)+L\left(t\right)-\;\sum_{w=1}^{N_{W}}\;W_{w}\left(t\right)-\;\sum_{m=1}^{N_{S}}\;S_{m}\left(t\right)-\sum_{j=1}^{N_{H}}H_{j}\left(t\right)-\;\sum_{i=1}^{N_{G}}\;{\tilde{P}}_{i}\left(t\right).$$

Defining the unit-wise available margin as

(31) $${\mathrm{room}}_{i}\left(t\right)=\left\{\begin{array}{cc} {\bar{P}}_{i}\left(t\right)-{\tilde{P}}_{i}\left(t\right), & \Delta (t)>0,\\ {\tilde{P}}_{i}\left(t\right)-{\underline{P}}_{i}\left(t\right), & \Delta (t)\leq 0, \end{array}\right.$$

the corrected dispatch is obtained by a single-pass proportional redistribution that respects each unit’s remaining ramp capacity:

(32) $$P_{i}\left(t\right)={\tilde{P}}_{i}\left(t\right)+\mathrm{sign}\;\left(\Delta (t)\right)\;\frac{{\mathrm{room}}_{i}\left(t\right)}{\sum_{j}{\mathrm{room}}_{j}\left(t\right)}\;\min\;\left(|\Delta \left(t\right)|,\;\sum_{j}{\mathrm{room}}_{j}\left(t\right)\right).$$

Because redistribution acts strictly within each unit’s feasible ramp interval, the capacity bounds in Equation (17) and the ramp limits in Equations (18) and (19) are preserved by construction. Any leftover mismatch, which can only occur when the aggregate ramp margin is insufficient to absorb |Δ(t)|, is recorded as an unresolved power-balance residual but never injected into the reward as a penalty. Across all reported PPO rollouts on both test systems, this residual remained below numerical tolerance (<$10^{-6}$ MW per hour), confirming that ramp margins were always sufficient for the studied benchmarks. Under extreme renewable fluctuations not represented in the training data, a non-zero residual would imply a temporary power imbalance that must be handled operationally (e.g., reserve activation or re-optimization); the Safety Layer therefore logs rather than hides such events. The procedure is deterministic, gradient-free, and executes in $O(N_{G})$ per timestep.

The PPO roll-out stores the raw actor output $a_{t}$ in the on-policy buffer, while the environment transitions use the corrected dispatch P(t) returned by the Safety Layer and the associated fuel cost. The policy gradient is therefore computed with respect to actions that produced feasible trajectories, encouraging the actor to approach the economically optimal region of the ramp-feasible set rather than delegating all constraint repair to the projection operator. Corrected actions are not written back into the buffer as surrogate labels, which preserves the standard on-policy PPO update. The complete pseudo-code of the Safety Layer is provided in Algorithm 1.
**Algorithm 1:** Safety Layer, feasibility repair for DED
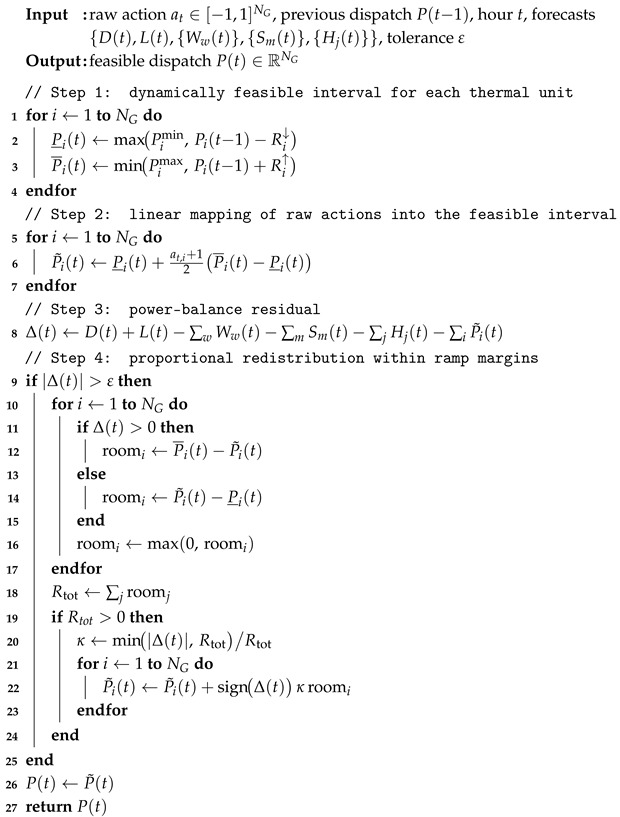


### 3.4. RLMPA: DQN-Guided Marine Predators Algorithm

The Marine Predators Algorithm (MPA) [16] is a nature-inspired metaheuristic that emulates the interaction between apex predators and prey through three hunting phases, governed by the step-size factor Π and the Fish Aggregating Devices (FADs) probability *F* [30]. Keeping Π and *F* fixed throughout the iterations frequently causes premature convergence in highly multimodal landscapes such as the valve-point-loaded cost surface of DED. To alleviate this, we equip MPA with a Deep Q-Network (DQN) that observes the search dynamics and adaptively perturbs Π and *F* at every iteration.

The RLMPA controller adjusts a low-dimensional discrete action space (five increments for each of *F* and Π), observed once per MPA iteration through a five-dimensional search-state vector. Deep Q-Networks are well suited to such small, discrete decision problems with delayed reward (cost improvement), whereas continuous policy-gradient methods (PPO, SAC, DDPG) are reserved for the hourly thermal dispatch in Stage 1. Alternatives such as policy gradients or evolutionary strategies on (F,Π) were not adopted because they add unnecessary variance for a two-parameter tuning task; the tabular *Q*-table variant embedded in the original MPA formulation [30] was replaced by DQN to improve generalization across the larger population matrices of the 20-unit system.

*Controller state:* At iteration *k*, the DQN observes a five-dimensional feature vector that summarizes the search progress, the population diversity of $X_{k}\in R^{N\times d}$, and the relative quality of the current best:

(33) $$s_{k}^{\mathrm{MPA}}=\left[\;\tau_{k},\;\rho_{k}^{\mathrm{conv}},\;D_{k},\;\eta_{k},\;0\;\right],$$

with

(34) $$\begin{array}{cc} \tau_{k} & =k/K, \end{array}$$

(35) $$\begin{array}{cc} \rho_{k}^{\mathrm{conv}} & =\max\;\left(0,\;\frac{f_{k-1}^{★}-f_{k}^{★}}{f_{k-1}^{★}}\right), \end{array}$$

(36) $$\begin{array}{cc} D_{k} & =\frac{1}{d}\;\sum_{j=1}^{d}\;\frac{\sigma_{j}\left(X_{k}\right)}{{\mathrm{range}}_{j}\left(X_{k}\right)}, \end{array}$$

(37) $$\begin{array}{cc} \eta_{k} & =\frac{f_{k}^{★}-\min_{i}f_{k}^{\left(i\right)}}{\max_{i}f_{k}^{\left(i\right)}-\min_{i}f_{k}^{\left(i\right)}+\varepsilon }. \end{array}$$

Here, $f_{k}^{★}$ is the best objective value at iteration *k*, $\sigma_{j}$ and ${\mathrm{range}}_{j}$ denote the coordinate-wise standard deviation and range of the population, and ε is a small constant that prevents division by zero in nearly converged swarms.

*Controller action:* The discrete action space $A_{\mathrm{DQN}}=\left\{+\;+,+,0,-,-\;-\right\}$ encodes five increments applied independently to *F* and Π with step vectors

(38) $$\begin{array}{cc} \Delta F & \in \{+0.02,\;+0.005,\;0,\;-0.005,\;-0.02\},\\ \Delta \Pi & \in \{+0.005,\;+0.001,\;0,\;-0.001,\;-0.005\}. \end{array}$$

After each update, the parameters are clipped to the operating ranges F∈[0.001,0.2] and Π∈[0.001,0.1].

*Reward:* A shaped improvement reward $r_{k}=\max\;\left(0,\;f_{k-1}^{★}-f_{k}^{★}\right)\cdot 10^{2}$ is fed back to the DQN, favoring configurations that produce monotonically decreasing best costs.

*Network and training:* The Q-network consists of two fully connected layers of 64 units each with ReLU activations, followed by a linear head of size 5. A target network $Q_{\theta^{-}}$ is synchronized every 50 updates. Transitions $\left(s_{k}^{\mathrm{MPA}},\;a_{k},\;r_{k},\;s_{k+1}^{\mathrm{MPA}}\right)$ are stored in a replay buffer of capacity 2000, and mini-batches of 64 samples are used for the Bellman update

(39) $$L_{\mathrm{DQN}}\left(\theta \right)=E\;\left[{\left(r_{k}+\gamma_{\mathrm{DQN}}\;\max_{a^{\prime }}Q_{\theta^{-}}\left(s_{k+1}^{\mathrm{MPA}},a^{\prime }\right)-Q_{\theta }\left(s_{k}^{\mathrm{MPA}},a_{k}\right)\right)}^{\;2}\right],$$

with discount factor $\gamma_{\mathrm{DQN}}=0.95$ and an ε-greedy exploration policy whose ε decays from 0.8 to 0.05 with rate 0.997. Two such DQN controllers operate in parallel (one dedicated to *F* and one to Π) and their parameters are updated online during MPA iterations, which keeps the controllers adaptive to the specific cost landscape of each optimization run.

*Three-phase MPA dynamics.* At each iteration, the population is updated through one of the three predator–prey phases of standard MPA, whose relative weighting is governed by the monotonically decreasing convergence factor

(40) $$\mathrm{CF}={\left(1-\frac{k}{K}\right)}^{2k/K},$$

where *k* and *K* denote the current and maximum iteration indices. The Brownian high-velocity-ratio phase dominates during k<K/3, a Brownian–Lévy transition phase during K/3≤k<2K/3, and a Lévy low-velocity-ratio exploitation phase during k≥2K/3. After each predator movement, the Safety Layer of Section 3.3 is re-applied as a feasibility repair operator, and the FADs eddy formation is triggered with probability *F* at the end of each iteration.

### 3.5. Two-Stage PPO–RLMPA Framework

The proposed Two-Stage PPO–RLMPA framework decomposes the DED optimization into two complementary stages, combining the macroscopic policy-level decision-making of PPO with the microscopic fine-tuning of the RLMPA module introduced in Section 3.4. The rationale is that PPO reaches a feasible near-optimal baseline in sub-second inference time, while RLMPA exploits this informed initialization to escape the local minima introduced by the valve-point-loading term of Equation (2).

#### 3.5.1. Stage 1–PPO with Safety Layer

At each scheduling hour *t*, the PPO actor observes $s_{t}$ from Equation (20), samples an action $a_{t}∼\pi_{\theta }\left(\cdot |s_{t}\right)$, and passes it through the Safety Layer described in Section 3.3. The resulting 24 h trajectory $x_{\mathrm{PPO}}={\left[P_{i}\left(t\right)\right]}_{i,t}$ is strictly feasible by construction. Because the actor is deterministic at inference, Stage 1 delivers a reproducible baseline in milliseconds and serves as the elite member for Stage 2.

#### 3.5.2. Stage 2–RLMPA Refinement

The PPO baseline is embedded as the top predator of the initial MPA population. The remaining N−1 individuals are drawn from a feasible initialization routine that respects both capacity and ramp bounds. MPA is then executed for K/2 iterations, with the DQN controllers of Section 3.4 dynamically adapting (F,Π) at every iteration. After each predator movement, the Safety Layer is reapplied to every candidate as the *repair operator*, which is critical because MPA’s Lévy-flight steps may otherwise propel candidates outside the ramp envelope.

**Figure 2:**
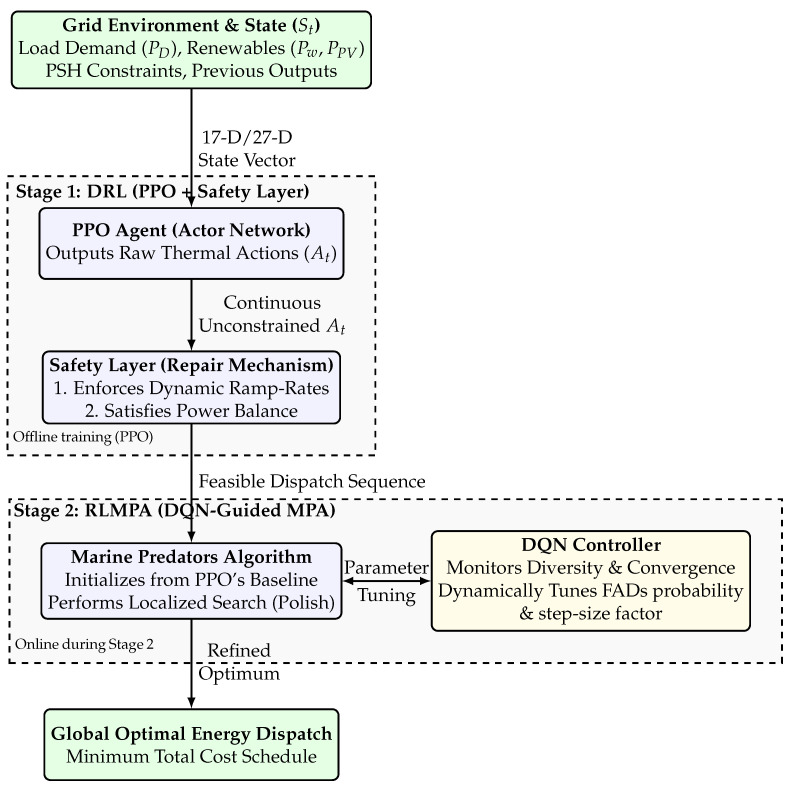
Flowchart of the proposed Two-Stage PPO–RLMPA framework for Dynamic Economic Dispatch. PPO weights are trained offline; deployed inference and optional RLMPA polishing operate on day-ahead or batch schedules.

Stage 1 guarantees feasibility and an informed starting region, while Stage 2 performs a localized exploration around the PPO baseline, thereby (i) avoiding the curse of dimensionality that affects random-start MPA in the 480-dimensional search space of Test System 2, (ii) breaking out of locally optimal valve-point peaks, and (iii) keeping the total runtime below 25 s per run. The overall pipeline is summarized in Algorithm 2, and its block diagram is shown in Figure 2.
**Algorithm 2:** Two-Stage PPO–RLMPA for DED
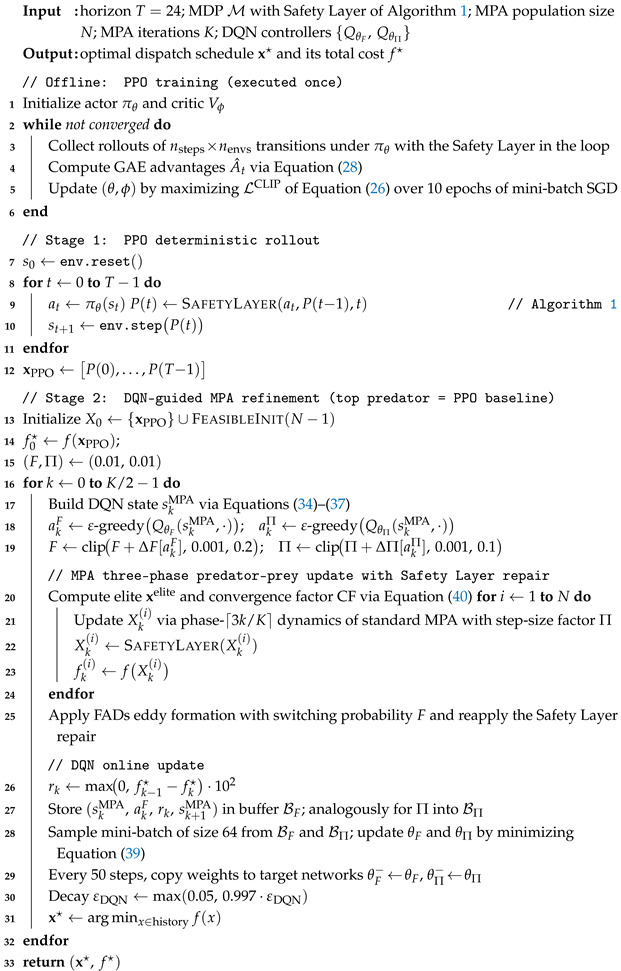


## 4. Simulation Results and Discussion

To evaluate the effectiveness and robustness of the Two-Stage PPO–RLMPA framework, it is tested on two different systems of Dynamic Economic Dispatch incorporating wind, solar PV, and pumped-storage hydro units. The proposed Two-Stage PPO–RLMPA framework is compared against classical MPA with a Repair mechanism, DQN-MPA with Repair, PSO with Repair, and the pure PPO with a Safety Layer.

The algorithms were executed for 30 independent runs to perform a statistically sound comparative analysis. Both test systems strictly enforce the valve-point loading effects, power balance, dynamic ramp-rate limits, and PSH operational constraints. Table 1 summarizes the simulation and hyperparameter settings. It is crucial to note that all techniques utilizing the custom Safety Layer or repair functions achieved a 0.00 average post-repair power-balance and ramp violation in the reported metrics; PPO rollouts additionally logged zero unresolved Safety Layer residual across all 30 runs on each system.

### 4.1. Test System 1

Test System 1 consists of 10 thermal generators, 1 equivalent wind power generating unit, 1 equivalent solar PV plant, and 1 PSH unit. The cost and operational parameters of the thermal generating units are summarized in Table 2. These parameters include minimum and maximum generation limits, quadratic cost coefficients, valve-point loading effects, and ramp-rate constraints. The data are adopted from [17] and rewritten using the notation of this study. These parameters are directly used in the objective function and constraint definitions. The scheduling horizon is divided into 24 h intervals. The valve-point loading effect is applied to all thermal generators.

The hourly load demand and ambient temperature profiles are given in Table 3. The load demand D(t) defines the power balance constraint at each time step, while the ambient temperature $T_{amb}\left(t\right)$ is used as an external input affecting renewable generation.

The temperature profile is particularly relevant for modeling solar photovoltaic (PV) output and is included in the state representation of the reinforcement learning agent. The renewable generation is driven by the time-varying solar irradiance and wind speed, as shown in Table 4. These data are taken from [32].

The solar irradiance G(t) determines the PV power output, while the wind speed $v_{w}\left(t\right)$ is used in the nonlinear wind power model. The rated capacity of the wind power unit is $W_{w}^{rated}=150$ MW. The cut-in, rated, and cut-out wind speeds are defined as $v_{w}^{\mathrm{in}}=4$ m/s, $v_{w}^{rated}=15$ m/s, and $v_{w}^{\mathrm{out}}=25$ m/s, respectively. The wind power conversion coefficient is denoted by $K_{w}=3.25$ [17].

Similarly, the rated capacity of the solar PV unit is $S_{m}^{rated}=150$ MW, with a conversion coefficient $K_{s}=3.5$. The standard solar radiation is taken as $G_{\mathrm{std}}=1000$ W/m^2^, while the reference radiation level is set to 150 W/m^2^ [17].

The PSH unit operates in both generating and pumping modes. During generation, the output power $H_{j}\left(t\right)$ satisfies $0\leq H_{j}\left(t\right)\leq 100$ MW, and the corresponding water discharge is given by

(41) $$Q^{\mathrm{psh}}\left(H_{j}\left(t\right)\right)=50+2H_{j}\left(t\right)$$

In pumping mode, the pumping power $U_{j}\left(t\right)$ is negative and satisfies $0\leq U_{j}\left(t\right)\leq 100$ MW. The water flow is modeled as

(42) $$Q^{\mathrm{psh}}\left(U_{j}\left(t\right)\right)=-200.$$

The reservoir is initialized at $V_{j}\left(0\right)=3000$ acre-ft and is required to return to the same level at the end of the scheduling horizon $V_{j}\left(T\right)=V_{j}\left(0\right)$. The water inflow and spillage effects are neglected in this study. Additionally, the PSH unit is restricted to operate at full pumping capacity when in pumping mode [17]. These profiles introduce additional variability and uncertainty into the dispatch problem, increasing its complexity. Overall, the combination of time-dependent demand and renewable inputs creates a highly dynamic and constrained optimization problem, which serves as a challenging benchmark for evaluating the proposed hybrid framework.

The performance of the proposed learning-based hybrid framework is evaluated through a series of numerical experiments on the considered Test System 1. Table 5 summarizes the comparative performance of the various optimization algorithms.

As observed in Table 5, the standalone PPO augmented with the Safety Layer immediately achieves a highly competitive best cost in fractions of a second, outperforming traditional metaheuristics in terms of computational time. By employing the Stage 2 RLMPA refinement on this PPO baseline, the algorithm successfully escapes remaining local optima, achieving a competitive minimum cost performance. This represents a substantial economic improvement over the benchmark established by CFCEP in [17].

**Figure 3:**
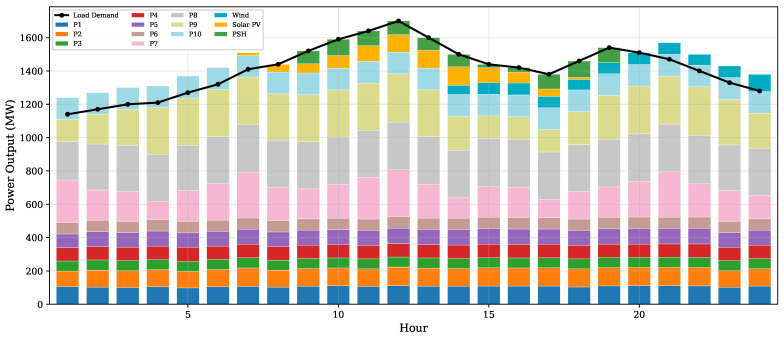
Optimal dynamic energy dispatch across the 24 h horizon for Test System 1 obtained by the proposed Two-Stage PPO–RLMPA framework.

Figure 3 illustrates the optimal dynamic energy dispatch for Test System 1 obtained by the proposed Two-Stage PPO–RLMPA framework over the 24 h horizon, showing coordinated operation among thermal units $P_{i}\left(t\right)$, renewable generation W(t) and S(t), and the PSH unit $\left(H_{j}\left(t\right),U_{j}\left(t\right)\right)$. Furthermore, Table 6 shows the optimal hourly dispatch schedule values for Test System 1 obtained by the proposed Two-Stage PPO–RLMPA framework.

### 4.2. Test System 2

In order to evaluate scalability on a higher-dimensional problem space, Test System 2 duplicates the generators of Test System 1, yielding $N_{G}=20$ thermal units, $N_{W}=2$ wind units, $N_{S}=2$ solar PV plants, and $N_{H}=2$ PSH units, with load demand doubled across all 24 intervals. This configuration tests dimensional scaling rather than a geographically distinct power system; evaluation on independent benchmarks with heterogeneous unit parameters and renewable profiles is identified as future work (Section 4.5). Since the state space expands to 27 dimensions and the action space to 20 continuous variables, classical metaheuristics suffer more heavily from the curse of dimensionality. Table 7 reports the performance results for Test System 2.

Remarkably, the pure PPO agent (Stage 1) alone discovers a highly superior policy, yielding a cost in near real time. By applying the Stage 2 RLMPA refinement, the methodology identifies the best cost of USD 737,348. When compared against the USD 771,132 benchmark reported by CFCEP [17], the Two-Stage PPO–RLMPA framework achieves a notable economic improvement.

**Figure 4:**
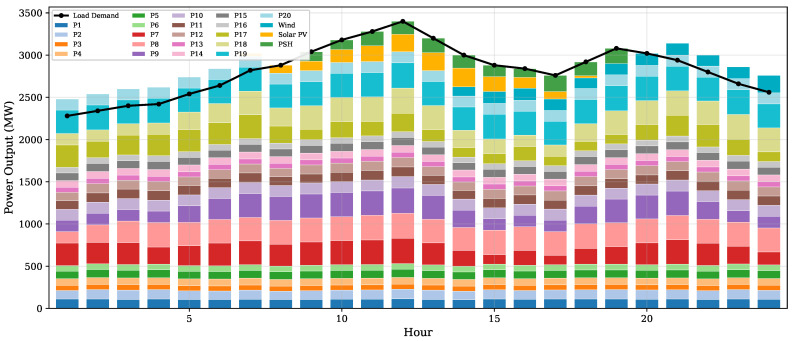
Optimal dynamic energy dispatch across the 24-h horizon for Test System 2 obtained by the proposed Two-Stage PPO–RLMPA framework.

Throughout all simulations in both Test System 1 and Test System 2, the Safety Layer and repair operator ensured that power-balance and ramp-rate constraints were satisfied in the final reported schedules (0.00 average violation after repair). Figure 4 illustrates the optimal dynamic energy dispatch obtained by the proposed framework across the 24 h horizon for Test System 2. Table 8 and Table 9 list the corresponding hourly dispatch values.

### 4.3. Ablation, Warm-Start, and Convergence

Table 10 interprets the existing baselines as an ablation of framework components on Test System 1. Random-start metaheuristics (MPA+Repair, DQN-MPA+Repair) remain more than USD 10,000 above PPO+SafetyLayer on average, confirming that PPO warm-start—not RLMPA search alone—delivers the primary economic gain. Stage 2 RLMPA adds a modest but consistent further reduction (∼0.3% mean cost on both systems) by polishing valve-point localities that PPO does not fully exploit.

**Figure 5:**
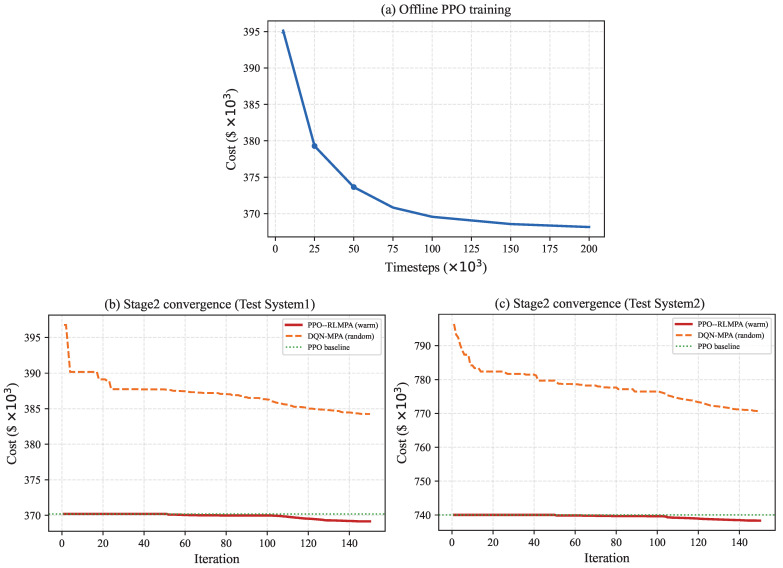
Convergence analysis of the Two-Stage PPO–RLMPA framework. (**a**) Offline PPO training. (**b**,**c**) Stage 2 RLMPA refinement with PPO elite initialization versus random-start DQN-MPA+Repair on Test Systems 1 and 2.

Figure 5 analyzes convergence in four panels. Panel (a) shows offline PPO training on Test System 1 (evaluation cost versus timesteps). Panels (b) and (c) compare Stage 2 best-cost trajectories for Test Systems 1 and 2, respectively, using an *equal* MPA iteration budget of K=150 for both curves: the solid line is PPO–RLMPA initialized from the PPO elite, the dashed line is DQN-MPA+Repair from a random feasible population, and the dotted horizontal line marks the Stage 1 PPO baseline. In both systems, warm-start refinement begins near the PPO cost and improves within the allotted iterations, whereas random-start search remains far above the PPO baseline over the same horizon. Panel (d) summarizes the offline/online execution modes. A single PPO training curve (Test System 1) is sufficient to demonstrate Stage 1 learning because the same algorithm and hyperparameter structure are used for both benchmarks; Stage 2 panels for both systems are included to show scalability of the warm-start benefit.

### 4.4. Computational Trade-Off

Sub-second Stage 1 inference supports fast what-if analysis or intra-day re-dispatch when RLMPA is skipped; the full pipeline (approximately 25 s on Test System 1 and 47 s on Test System 2 in our experiments) trades additional computation for 0.3–4.4% cost improvement relative to Stage 1 alone or to CFCEP, which is acceptable for offline day-ahead scheduling but not for sub-second SCED cycles.

### 4.5. Discussion

The comparative results on both benchmark systems reveal several complementary insights regarding the economic, computational, and statistical behavior of the proposed Two-Stage PPO–RLMPA framework. The economic advantages become exceptionally pronounced in the larger and more complex Test System 2: whereas the CFCEP algorithm previously established a benchmark best cost of USD 771,132 [17], the proposed framework achieves a best cost of USD 737,348, corresponding to a direct saving of USD 33,631 (approximately 4.4% cost reduction) over the entire 24 h scheduling horizon. The method is also highly robust, with a mean cost of USD 738,152 over 30 independent runs—significantly lower than the absolute best costs ever reported by pure PSO or baseline MPA methodologies—which underscores the efficacy of using a continuous-action PPO agent to rapidly navigate the high-dimensional search space and bypass the curse of dimensionality, while the RLMPA refinement efficiently resolves the localized non-convexities introduced by the valve-point loading effects of the 20 thermal generators.

Beyond raw cost, the sub-second inference of the PPO agent equipped with the Safety Layer already outperforms all population-based baselines in both solution quality and runtime, suggesting that embedding feasibility projection directly inside the MDP acts as a strong inductive bias on its own. The RLMPA stage then contributes a consistent but modest additional improvement of approximately 0.3% on both Test Systems 1 and 2, indicating that the DQN-guided MPA primarily mitigates the residual local-optimum risk introduced by valve-point effects rather than performing global exploration; the two stages are complementary, with PPO handling global navigation and RLMPA performing targeted local polishing. Metaheuristic baselines exhibit non-zero cost standard deviation across seeds (Table 5), whereas PPO inference is deterministic once trained (Std =0), which aids reproducibility for operators. Hyperparameters for PPO and DQN were fixed across both systems; the gap between MPA+Repair and DQN-MPA+Repair indicates moderate sensitivity of standalone metaheuristics to adaptive (F,Π) control, while the full framework remained stable on the 10- and 20-unit cases without retuning.

### 4.6. Limitations

The study assumes deterministic renewable forecasts, a fixed fleet availability, and does not model unit outages, minimum down-time, or maintenance derates—extensions that would require time-varying $P_{i}^{\max}$ and additional binary commitment logic. Test System 2 is a structured scale-up of Test System 1 rather than an independent network; broader validation on heterogeneous benchmarks is left to future work. Natural extensions include stochastic or scenario-based forecasts, multi-horizon rolling dispatch, and evaluation under real-time market signals.

## 5. Conclusions

This paper presented the Two-Stage PPO–RLMPA framework for Dynamic Economic Dispatch in renewable-integrated power systems. The main contributions are fourfold: (i) an MDP formulation with a deterministic Safety Layer that enforces ramp and power-balance feasibility at every hour without penalty tuning; (ii) a PPO policy that achieves competitive day-ahead costs with sub-second inference after offline training; (iii) a DQN-guided RLMPA refinement stage that polishes the PPO baseline around valve-point local optima; and (iv) comprehensive validation on ten- and twenty-unit Basu benchmarks with statistical reporting over 30 independent runs.

On Test Systems 1 and 2, the framework attained best costs of USD 368,763 and USD 737,348, improving upon the CFCEP reference by approximately 1.1% and 4.4%, with zero post-repair constraint violations. Ablation through existing baselines showed that PPO warm-start accounts for the dominant economic gain, while RLMPA adds a modest but repeatable Stage 2 improvement (∼0.3% mean cost). Stage 1 alone is appropriate when sub-second schedules are required; the full pipeline is justified for offline day-ahead planning when additional computational budget (∼20 s) is acceptable.

Future work will address stochastic renewable forecasts, time-varying unit availability (outages and minimum down-time), multi-horizon rolling dispatch, independent benchmark systems with heterogeneous generator fleets, and optional LP/QP-based safety projections for tighter ramp margins under extreme operating conditions.

## Figures and Tables

**Table 1:** Simulation and algorithm hyperparameters used in all experiments.

Category	Parameter	Value
Problem	Scheduling horizon *T*	24 h
	Test System 1/2 thermal units $N_{G}$	10/20
	Independent runs per method	30
	Random seed for run *r*	42+r
PPO (Stage 1)	Parallel rollout environments	4
	Steps per rollout $n_{\mathrm{steps}}$	192
	Mini-batch size	48
	PPO epochs per update	10
	Learning rate	$3\times 10^{-4}$
	Training timesteps (TS1/TS2)	150,000/200,000
MPA/RLMPA (Stage 2)	Population size *N*	50
	MPA iterations (standalone baselines)	300
	RLMPA iterations (after PPO warm-start)	150
	Repair operator	repair_solution
DQN meta-controller	Hidden layers	64×64
	Learning rate	$1\times 10^{-3}$
	Replay buffer capacity	2000
	Discount $\gamma_{\mathrm{DQN}}$	0.95

**Table 2:** Thermal generator parameters for Test System 1 (adapted from [17]).

Unit	$P_{i}^{\min}$	$P_{i}^{\max}$	$\alpha_{i}$	$\beta_{i}$	$\chi_{i}$	$\delta_{i}$	$\zeta_{i}$	$R_{i}^{↑}$	$R_{i}^{↓}$
	(MW)	(MW)	($/h)	($/MWh)	($/MW^2^h)	($/h)	(rad/MW)	(MW/h)	(MW/h)
1	36	114	94.705	6.73	0.00690	100	0.084	40	40
2	36	114	94.705	6.73	0.00690	100	0.084	40	40
3	60	120	309.540	7.07	0.02028	100	0.084	40	40
4	80	190	369.030	8.18	0.00942	150	0.063	60	60
5	47	97	148.890	5.35	0.01140	120	0.077	30	30
6	68	140	222.330	8.05	0.01142	100	0.084	50	50
7	110	300	287.710	8.03	0.00357	200	0.042	80	80
8	135	300	391.980	6.99	0.00492	200	0.042	80	80
9	135	300	455.760	6.60	0.00573	200	0.042	80	80
10	130	300	722.820	12.90	0.00605	200	0.042	80	80

**Table 3:** Hourly load demand and ambient temperature for Test System 1 [17].

*t*	D(t)	$T_{\mathrm{amb}}\left(t\right)$	*t*	D(t)	$T_{\mathrm{amb}}\left(t\right)$	*t*	D(t)	$T_{\mathrm{amb}}\left(t\right)$
(h)	(MW)	°C	(h)	(MW)	°C	(h)	(MW)	°C
1	1140	23	9	1520	27	17	1380	26
2	1170	23	10	1590	28	18	1460	25
3	1200	24	11	1640	29	19	1540	24
4	1210	25	12	1700	29	20	1510	24
5	1270	25	13	1600	29	21	1470	24
6	1320	25	14	1500	28	22	1400	24
7	1410	26	15	1440	28	23	1330	23
8	1440	27	16	1420	27	24	1280	23

**Table 4:** Forecast solar irradiance and wind speed profiles [32].

*t*	G(t)	$v_{w}\left(t\right)$	*t*	G(t)	$v_{w}\left(t\right)$	*t*	G(t)	$v_{w}\left(t\right)$
(h)	(W/m^2^)	(m/s)	(h)	(W/m^2^)	(m/s)	(h)	(W/m^2^)	(m/s)
1	0	3.5	9	375	3.8	17	291	9.7
2	0	3.6	10	503	3.7	18	86	9.2
3	0	1.5	11	617	2.0	19	0	9.6
4	0	1.4	12	686	0.6	20	0	10.0
5	0	0.1	13	703	0.4	21	0	10.0
6	0	1.8	14	736	8.4	22	0	9.5
7	111	1.3	15	586	9.9	23	0	9.9
8	311	2.2	16	425	10.1	24	0	12.6

**Table 5:** Performance comparison on Test System 1 (30 independent runs). Mean ± standard deviation reported where applicable.

Method	Mean ± Std (USD)	Best (USD)	Worst (USD)	Avg. Time (s)
MPA+Repair	381,501 ± 1031	379,536	383,506	30.9
DQN-MPA+Repair	380,607 ± 1381	378,346	382,958	56.4
PSO+Repair	383,732 ± 1217	381,545	387,009	12.0
PPO+SafetyLayer	370,196 ± 0	370,196	370,196	0.0
PPO–RLMPA (Proposed)	369,202 ± 215	368,763	369,665	25.0
CFCEP [17]	372,738	372,735	372,744	N/A

**Table 6:** Optimal hourly dispatch schedule obtained by the proposed Two-Stage PPO–RLMPA framework on Test System 1. All power values are in MW.

*t*	$P_{1}\left(t\right)$	$P_{2}\left(t\right)$	$P_{3}\left(t\right)$	$P_{4}\left(t\right)$	$P_{5}\left(t\right)$	$P_{6}\left(t\right)$	$P_{7}\left(t\right)$	$P_{8}\left(t\right)$	$P_{9}\left(t\right)$	$P_{10}\left(t\right)$	$P_{\mathrm{th}}\left(t\right)$	W(t)	S(t)	$P_{\mathrm{psh}}\left(t\right)$	$P_{\mathrm{tot}}\left(t\right)$	D(t)
1	105.23	93.05	60.48	80.03	82.84	68.16	255.12	229.81	135.12	130.16	1240.00	0.00	0.00	−100.00	1240.00	1140
2	102.73	102.46	60.23	80.00	89.43	68.05	182.49	276.06	178.40	130.16	1270.00	0.00	0.00	−100.00	1270.00	1170
3	100.41	100.90	60.22	80.45	87.04	68.20	178.49	276.87	217.13	130.30	1300.00	0.00	0.00	−100.00	1300.00	1200
4	104.39	103.84	60.08	80.37	90.45	68.17	110.28	280.04	282.29	130.08	1310.00	0.00	0.00	−100.00	1310.00	1210
5	97.90	98.16	61.21	85.09	85.42	68.93	183.92	273.34	283.25	132.77	1370.00	0.00	0.00	−100.00	1370.00	1270
6	104.08	104.51	60.02	80.18	87.74	68.11	221.92	278.75	283.84	130.84	1420.00	0.00	0.00	−100.00	1420.00	1320
7	106.21	112.04	60.12	81.02	90.61	68.12	275.25	285.17	284.50	130.27	1493.31	0.00	16.69	−100.00	1510.00	1410
8	102.49	102.43	60.19	80.18	88.28	68.21	198.51	283.88	278.87	130.09	1393.12	0.00	46.88	0.00	1440.00	1440
9	108.15	105.91	60.15	80.07	89.75	68.44	179.06	282.79	282.68	130.11	1387.12	0.00	56.53	76.35	1520.00	1520
10	110.79	107.04	60.07	80.12	88.86	68.42	204.95	282.50	283.33	130.07	1416.13	0.00	76.02	97.86	1590.00	1590
11	106.39	105.46	60.19	80.67	90.21	68.32	250.13	281.46	284.18	130.55	1457.55	0.00	93.48	88.97	1640.00	1640
12	110.76	111.96	60.27	80.26	93.49	68.42	281.02	286.41	290.38	130.36	1513.33	0.00	103.93	82.74	1700.00	1700
13	108.06	108.85	60.33	80.12	91.24	68.38	202.52	284.91	282.54	130.23	1417.18	0.00	106.50	76.32	1600.00	1600
14	108.18	106.74	60.03	80.49	92.05	68.13	122.76	284.24	205.72	130.22	1258.56	54.71	111.23	75.49	1500.00	1500
15	108.94	110.03	60.08	80.45	93.99	68.36	183.35	289.63	135.35	130.27	1260.45	69.63	88.56	21.36	1440.00	1440
16	108.71	109.23	60.29	80.04	93.30	68.19	183.60	286.00	135.87	130.54	1255.77	71.83	64.07	28.33	1420.00	1420
17	108.76	110.58	60.03	80.33	91.10	68.11	110.82	283.68	135.28	131.16	1179.84	67.49	43.76	88.91	1380.00	1380
18	103.21	109.36	60.07	80.06	89.56	68.37	165.67	280.18	199.01	130.13	1285.61	62.33	12.90	99.15	1460.00	1460
19	109.62	110.58	60.08	80.22	91.67	68.39	183.54	286.67	262.44	130.35	1383.56	66.43	0.00	90.01	1540.00	1540
20	110.52	110.20	60.16	80.09	93.74	68.12	213.02	286.96	286.36	130.10	1439.28	70.72	0.00	0.00	1510.00	1510
21	110.51	110.77	60.70	80.17	92.24	68.07	271.54	287.14	287.48	130.65	1499.28	70.72	0.00	−100.00	1570.00	1470
22	110.32	111.02	60.08	80.09	93.68	68.15	198.94	291.06	291.00	130.27	1434.61	65.39	0.00	−100.00	1500.00	1400
23	100.72	100.58	60.04	80.16	87.52	68.31	184.02	274.73	273.70	130.59	1360.37	69.63	0.00	−100.00	1430.00	1330
24	108.50	105.48	60.04	80.14	90.15	68.11	141.38	282.77	209.60	130.51	1276.68	103.32	0.00	−100.00	1380.00	1280

**Table 7:** Performance comparison on Test System 2 (30 independent runs). Mean ± standard deviation reported where applicable.

Method	Mean ± Std (USD)	Best (USD)	Worst (USD)	Avg. Time (s)
MPA+Repair	769,688 ± 2012	765,163	774,514	51.9
DQN-MPA+Repair	768,725 ± 2052	762,984	771,824	84.5
PSO+Repair	772,369 ± 2888	766,082	777,539	29.7
PPO+SafetyLayer	740,031 ± 0	740,031	740,031	0.0
PPO–RLMPA (Proposed)	738,152 ± 371	737,348	738,746	47.1
CFCEP [17]	771,132	771,132	771,132	N/A

**Table 8:** Optimal hourly dispatch schedule obtained by the proposed Two-Stage PPO–RLMPA framework on Test System 2—thermal units $P_{1}$ through $P_{14}$ (MW).

*t*	$P_{1}\left(t\right)$	$P_{2}\left(t\right)$	$P_{3}\left(t\right)$	$P_{4}\left(t\right)$	$P_{5}\left(t\right)$	$P_{6}\left(t\right)$	$P_{7}\left(t\right)$	$P_{8}\left(t\right)$	$P_{9}\left(t\right)$	$P_{10}\left(t\right)$	$P_{11}\left(t\right)$	$P_{12}\left(t\right)$	$P_{13}\left(t\right)$	$P_{14}\left(t\right)$
1	110.86	100.52	60.35	80.05	87.67	68.25	265.49	135.21	135.23	130.04	100.35	100.26	60.46	80.11
2	110.86	110.95	61.03	82.08	94.50	69.03	250.72	209.85	137.20	130.77	110.57	110.66	61.96	81.69
3	103.97	112.61	63.40	80.30	90.13	68.18	259.21	256.94	135.36	130.00	113.36	104.73	60.03	80.09
4	110.66	110.98	60.30	80.03	94.37	68.28	200.71	291.31	135.23	130.16	110.73	111.03	60.47	80.50
5	103.46	104.37	60.21	80.82	88.96	68.23	236.24	277.40	194.00	130.27	110.19	102.94	60.37	81.22
6	103.36	102.96	60.04	80.48	88.71	68.16	270.10	278.76	247.57	130.35	109.94	103.09	61.41	80.10
7	106.89	109.33	60.47	82.02	89.71	68.47	281.67	278.21	282.24	130.86	113.63	106.39	60.19	81.12
8	104.87	97.80	60.03	80.20	89.72	68.22	258.45	282.91	282.47	130.18	104.26	98.96	60.04	80.02
9	104.78	106.02	60.11	80.46	90.85	68.07	277.26	283.89	283.96	130.38	106.34	105.92	60.21	80.06
10	108.97	107.66	60.23	80.09	91.99	68.27	283.55	284.05	286.55	130.29	109.14	109.80	60.09	80.32
11	109.11	109.06	60.33	80.19	92.22	68.20	290.75	292.24	288.75	130.18	110.34	110.11	60.12	80.20
12	113.40	113.23	60.25	80.08	95.78	68.51	298.59	297.66	297.49	136.58	113.31	112.86	60.81	80.33
13	105.06	110.97	60.16	80.01	89.82	68.25	261.86	279.65	282.14	131.22	104.21	105.88	60.21	80.04
14	101.94	101.24	60.03	80.05	87.32	68.18	186.19	273.78	203.98	130.80	101.42	101.61	61.35	80.00
15	110.44	109.95	60.65	81.27	94.05	68.08	110.49	291.56	135.20	130.05	109.85	109.97	60.46	80.39
16	106.05	105.70	60.11	80.06	90.86	68.07	173.79	283.08	135.30	130.11	109.93	105.77	60.07	80.01
17	111.27	106.75	60.04	80.21	90.77	68.28	110.38	281.71	135.45	130.46	106.73	108.96	60.20	80.02
18	111.06	110.46	60.01	80.10	94.42	68.18	184.78	292.79	208.33	130.82	110.52	110.77	60.48	80.16
19	110.59	111.51	60.16	80.20	93.65	68.16	206.21	283.17	279.71	130.51	110.42	110.49	60.34	80.36
20	107.35	110.49	60.13	80.56	91.65	68.24	258.23	283.89	281.90	130.34	106.78	111.53	60.14	80.03
21	111.20	109.50	60.34	80.38	93.53	68.12	288.68	289.51	288.54	130.17	109.35	108.84	60.64	80.76
22	104.74	105.45	60.13	80.39	89.20	68.26	263.42	282.92	209.35	130.17	112.67	108.44	60.20	80.39
23	111.31	110.73	60.04	80.14	95.01	68.10	209.98	286.80	135.28	130.43	110.93	110.45	60.14	80.35
24	106.58	106.88	60.37	80.94	91.51	68.56	152.92	284.92	137.49	131.83	108.85	107.03	61.08	81.43

**Table 9:** Optimal hourly dispatch schedule for Test System 2 (continued from Table 8)—thermal units $P_{15}$ through $P_{20}$ and system totals (MW).

*t*	$P_{15}\left(t\right)$	$P_{16}\left(t\right)$	$P_{17}\left(t\right)$	$P_{18}\left(t\right)$	$P_{19}\left(t\right)$	$P_{20}\left(t\right)$	$P_{\mathrm{th}}\left(t\right)$	W(t)	S(t)	$P_{\mathrm{psh}}\left(t\right)$	$P_{\mathrm{tot}}\left(t\right)$	D(t)
1	88.72	68.05	264.75	135.05	278.41	130.18	2480.00	0.00	0.00	−200.00	2480.00	2280.00
2	94.97	68.45	192.28	137.37	293.38	131.67	2540.00	0.00	0.00	−200.00	2540.00	2340.00
3	90.69	68.00	234.72	135.29	282.89	130.11	2600.00	0.00	0.00	−200.00	2600.00	2400.00
4	94.36	68.63	252.92	135.80	293.06	130.46	2620.00	0.00	0.00	−200.00	2620.00	2420.00
5	88.80	68.39	259.59	209.45	284.90	130.18	2740.00	0.00	0.00	−200.00	2740.00	2540.00
6	88.11	68.71	255.28	228.64	284.23	130.00	2840.00	0.00	0.00	−200.00	2840.00	2640.00
7	91.72	68.22	281.88	280.12	283.18	130.30	2986.62	0.00	33.38	−200.00	3020.00	2820.00
8	90.65	68.31	203.00	214.52	281.34	130.27	2786.23	0.00	93.77	0.00	2880.00	2880.00
9	90.55	68.17	123.11	280.61	284.17	130.12	2815.03	0.00	113.06	111.90	3040.00	3040.00
10	92.95	68.27	189.51	286.39	285.39	130.14	2913.61	0.00	152.03	114.36	3180.00	3180.00
11	93.45	68.14	170.85	290.75	288.02	130.30	2923.32	0.00	186.95	169.73	3280.00	3280.00
12	94.71	68.59	217.28	299.76	298.48	130.99	3038.70	0.00	207.86	153.44	3400.00	3400.00
13	89.63	68.17	139.80	284.64	284.74	130.14	2816.61	0.00	213.01	170.38	3200.00	3200.00
14	86.27	68.19	110.02	206.67	275.33	130.17	2514.54	109.43	222.46	153.58	3000.00	3000.00
15	93.75	68.02	118.29	174.97	291.70	130.24	2429.38	139.26	177.12	134.24	2880.00	2880.00
16	91.40	68.28	166.81	135.63	282.91	130.23	2464.18	143.65	128.14	104.03	2840.00	2840.00
17	90.26	68.18	110.34	135.35	279.83	130.17	2345.38	134.97	87.52	192.13	2760.00	2760.00
18	95.04	68.38	110.11	209.96	287.41	130.27	2604.04	124.67	25.80	165.50	2920.00	2920.00
19	94.12	68.08	110.20	283.12	295.45	130.13	2766.59	132.86	0.00	180.55	3080.00	3080.00
20	92.67	68.16	184.10	285.05	287.27	130.04	2878.55	141.45	0.00	0.00	3020.00	3020.00
21	93.09	68.14	246.55	289.26	291.34	130.61	2998.55	141.45	0.00	−200.00	3140.00	2940.00
22	88.72	68.14	264.50	278.41	283.55	130.16	2869.22	130.78	0.00	−200.00	3000.00	2800.00
23	94.65	68.37	190.22	293.94	293.77	130.12	2720.74	139.26	0.00	−200.00	2860.00	2660.00
24	91.54	68.30	112.66	284.01	285.01	131.45	2553.37	206.63	0.00	−200.00	2760.00	2560.00

**Table 10:** Ablation interpretation on Test System 1 (30 runs): which component each baseline removes.

Configuration	Method	Mean Cost (USD)
No PPO (random feasible start)	MPA+Repair	381,501
+ DQN meta-control	DQN-MPA+Repair	380,607
PPO + Safety Layer (no Stage 2)	PPO+SafetyLayer	370,196
Full two-stage framework	PPO-RLMPA	369,202

## Data Availability

The raw data supporting the conclusions of this article will be available by the author on request.

## Abbreviations

*t*	Time interval index
*i*	Index of thermal generating units
*w*	Index of wind generation units
*m*	Index of photovoltaic units
*j*	Index of pumped-storage hydro units
r,s	Indices used in transmission loss calculation
*T*	Total number of scheduling intervals
$N_{G}$	Number of thermal generators
$N_{W}$	Number of wind units
$N_{S}$	Number of photovoltaic units
$N_{H}$	Number of pumped-storage hydro units
$N_{T}$	Total number of generating units
$P_{i}\left(t\right)$	Power output of thermal generator *i* at time *t* (MW)
$W_{w}\left(t\right)$	Power output of wind unit *w* at time *t* (MW)
$S_{m}\left(t\right)$	Power output of PV unit *m* at time *t* (MW)
$H_{j}\left(t\right)$	Generating power of PSH unit *j* at time *t* (MW)
$U_{j}\left(t\right)$	Pumping power of PSH unit *j* at time *t* (MW)
$V_{j}\left(t\right)$	Reservoir storage volume of PSH unit *j* at time *t*
D(t)	Load demand at time *t* (MW)
L(t)	Transmission loss at time *t* (MW)
$c_{w}^{W}$	Cost coefficient of wind generation unit *w*
$c_{m}^{S}$	Cost coefficient of PV generation unit *m*
$P_{i}^{\min}$	Minimum output limit of thermal generator *i* (MW)
$P_{i}^{\max}$	Maximum output limit of thermal generator *i* (MW)
$R_{i}^{↑}$	Ramp-up limit of thermal generator *i* (MW/h)
$R_{i}^{↓}$	Ramp-down limit of thermal generator *i* (MW/h)
$V_{j}^{\min}$	Minimum reservoir volume of PSH unit *j*
$V_{j}^{\max}$	Maximum reservoir volume of PSH unit *j*
$\alpha_{i}$	Constant fuel cost coefficient of thermal generator *i*
$\beta_{i}$	Linear fuel cost coefficient of thermal generator *i*
$\chi_{i}$	Quadratic fuel cost coefficient of thermal generator *i*
$\delta_{i}$	Valve-point loading coefficient of generator *i*
$\zeta_{i}$	Valve-point ripple coefficient of thermal generator *i*
$B_{rs}$	Transmission loss coefficient matrix element
$B_{r0}$	Transmission loss coefficient vector element
$B_{00}$	Constant transmission loss coefficient
$v_{w}\left(t\right)$	Wind speed at unit *w* and time *t* (m/s)
$v_{w}^{\mathrm{in}}$	Cut-in wind speed of unit *w* (m/s)
$v_{w}^{\mathrm{rated}}$	Rated wind speed of unit *w* (m/s)
$v_{w}^{\mathrm{out}}$	Cut-out wind speed of unit *w* (m/s)
$W_{w}^{\mathrm{rated}}$	Rated output power of wind unit *w* (MW)
$a_{w},b_{w},c_{w}$	Wind power curve coefficients
$S_{m}^{\mathrm{rated}}$	Rated PV output power of unit *m* (MW)
G(t)	Solar irradiance at time *t* (W/m^2^)
$G_{\mathrm{std}}$	Standard-test-condition solar irradiance (1000 W/m^2^)
$T_{\mathrm{amb}}\left(t\right)$	Ambient temperature at time *t* (°C)
$T_{\mathrm{ref}}$	Reference temperature (°C)
κ	PV temperature derating coefficient (κ>0), per °C
$\phi_{j}$	Pumping conversion coefficient of PSH unit *j*
$\psi_{j}$	Generation conversion coefficient of PSH unit *j*
*J*	Total generation cost over scheduling horizon
$s_{t}$	System state at time *t*
$r_{t}$	Reward at time *t*
θ	Policy network parameters
$\rho_{t}\left(\theta \right)$	Probability ratio in PPO update
ϵ	PPO clipping parameter
γ	Discount factor
*k*	Iteration index of MPA
*K*	Maximum number of iterations
$\tau_{k}$	Normalized iteration ratio (k/K)
$\rho_{k}^{\mathrm{conv}}$	Convergence indicator
$D_{k}$	Population diversity metric, $D_{k}=\frac{1}{d}\sum_{j=1}^{d}\sigma_{j}\left(X_{k}\right)/{\mathrm{range}}_{j}\left(X_{k}\right)$
$\eta_{k}$	Rank-based quality indicator
$s_{k}^{\mathrm{MPA}}$	State vector for DQN controller
$Q_{\theta }\left(s,d\right)$	Action-value function
$F_{\mathrm{ADs}}$	Switching probability in MPA
Π	Step-size parameter in MPA
$f_{k}^{*}$	Best objective value at iteration *k*
$x^{\mathrm{PPO}}$	Dispatch trajectory generated by PPO
$x^{*}$	Final optimized solution
$P_{th}\left(t\right)$	Total thermal generation at time *t* (MW)
W(t)	Total wind power generation at time *t* (MW)
S(t)	Total PV power generation at time *t* (MW)
$P_{psh}\left(t\right)$	Net PSH power at time *t*, where $P_{psh}\left(t\right)=H_{j}\left(t\right)$ (generation) and $P_{psh}\left(t\right)=-U_{j}\left(t\right)$ (pumping) (MW)
$P_{\mathrm{tot}}\left(t\right)$	Total generated power supplied to the grid at time *t* $P_{\mathrm{tot}}\left(t\right)=P_{\mathrm{th}}\left(t\right)+W\left(t\right)+S\left(t\right)+\max\left(0,\;P_{\mathrm{psh}}\left(t\right)\right)$
M	Markov Decision Process tuple (S,A,P,R,γ)
S	State space of the MDP
A	Action space of the MDP
P	Transition kernel induced by grid dynamics and Safety Layer
R	Scalar reward function
$\tilde{\left(\cdot \right)}$	Normalized quantity (scaled by nominal capacity)
ρ(t)	Required thermal share at time *t*, $\left(D-W-S-H\right)/\;\sum_{i}P_{i}^{\max}$
${\underline{P}}_{i}\left(t\right)$	Lower bound of the dynamically feasible interval of unit *i* at time *t*
${\bar{P}}_{i}\left(t\right)$	Upper bound of the dynamically feasible interval of unit *i* at time *t*
${\tilde{P}}_{i}\left(t\right)$	Provisional (pre-repair) dispatch of unit *i* at time *t*
${\hat{P}}_{i}\left(t-1\right)$	Normalized previous-hour output of thermal unit *i*, ${\hat{P}}_{i}\left(t-1\right)=\left(P_{i}\left(t-1\right)-P_{i}^{\min}\right)/\left(P_{i}^{\max}-P_{i}^{\min}\right)$
$C_{i}\left(\cdot \right)$	Fuel cost function of thermal unit *i*
$C_{\mathrm{norm}}$	Reward normalization constant ($10^{4}$)
$\pi_{\theta }$	Stochastic policy parameterized by θ
$V_{\phi }$	Value function parameterized by ϕ
ϕ	Critic network parameters
${\hat{A}}_{t}$	Generalized Advantage Estimation (GAE) at time *t*
$\delta_{t}$	Temporal-difference residual at time *t*
$L^{\mathrm{CLIP}}\left(\theta \right)$	Clipped surrogate PPO objective
L(θ,ϕ)	Total PPO loss (policy + value + entropy)
$H(\pi_{\theta })$	Entropy of the stochastic policy
$R_{t}$	Discounted return at time *t*
$c_{1}$	Value-loss coefficient in the PPO total loss
$c_{2}$	Entropy bonus coefficient in the PPO total loss
λ	GAE smoothing parameter
$n_{\mathrm{steps}}$	Number of environment steps per PPO rollout
$n_{\mathrm{envs}}$	Number of parallel environments used during PPO training
Δ(t)	Power-balance residual at time *t* (pre-repair)
${\mathrm{room}}_{i}\left(t\right)$	Available ramp margin of unit *i* used by the Safety Layer
ε	Numerical tolerance for the mismatch check
$X_{k}$	MPA population matrix at iteration *k*, $X_{k}\;\in \;R^{N\times d}$
*N*	MPA population size (number of search agents)
*d*	Dimensionality of a candidate solution, $d\;=\;N_{G}\;\cdot \;T$
$\sigma_{j}\left(X_{k}\right)$	Coordinate-wise standard deviation of population $X_{k}$ along axis *j*
${\mathrm{range}}_{j}\left(X_{k}\right)$	Coordinate-wise range of population $X_{k}$ along axis *j*
$f_{k}^{\left(i\right)}$	Objective value of the *i*-th candidate at iteration *k*
$r_{k}$	Shaped improvement reward fed to the DQN controller
$\gamma_{\mathrm{DQN}}$	Discount factor used in the DQN Bellman update
$Q_{\theta }$	Policy (online) Q-network of the DQN controller
$Q_{\theta^{-}}$	Target Q-network synchronized every 50 updates
$\varepsilon_{\mathrm{DQN}}$	ε-greedy exploration rate of the DQN
$B_{F},\;B_{\Pi }$	Replay buffers of the FADs and step-size DQN controllers
$L_{\mathrm{DQN}}$	Mean-squared Bellman loss of the DQN
ΔF[·]	Action-dependent increment applied to the FADs probability *F*
ΔΠ[·]	Action-dependent increment applied to the step-size factor Π
$a_{k}^{F},\;a_{k}^{\Pi }$	Discrete DQN actions controlling *F* and *P* at iteration *k*
$x^{\mathrm{elite}}$	Top-predator (elite) solution of MPA
CF	Convergence factor of MPA, $\mathrm{CF}={\left(1-k/K\right)}^{2k/K}$
FEASIBLEINIT(·)	Feasible initialization routine (ramp & capacity aware)
SAFETYLAYER(·)	Deterministic repair operator (Algorithm 1)
